# Reducing/Recycling Product Packaging Materials and Implementing Sustainable Development Goal Initiatives in Orthopedic Artificial Joint Manufacturing and Sales Companies: A Questionnaire-Based Survey

**DOI:** 10.7759/cureus.74556

**Published:** 2024-11-27

**Authors:** Haruo Kawamura, Tomofumi Nishino, Hajime Mishima

**Affiliations:** 1 Orthopedic Surgery, Kenpoku Medical Center Takahagi Kyodo Hospital, Ibaraki, JPN; 2 Orthopedic Surgery, Institute of Medicine, Tsukuba, JPN; 3 Orthopedic Surgery, University of Tsukuba, Tsukuba, JPN

**Keywords:** artificial joint, manufacturer, packaging material, recycling, sustainability, waste

## Abstract

Background and objective

Orthopedic surgery, particularly joint replacement, involves the use of many implants, resulting in a large amount of product packaging waste. To date, no study has surveyed artificial joint manufacturers on the recycling and reduction of packaging materials and their Sustainable Development Goal (SDG) initiatives. This questionnaire survey aimed to identify the current status of orthopedic artificial joint manufacturers in terms of implementing SDG initiatives.

Material and methods

The questionnaire survey involved 16 companies that sell artificial joints in Japan. The questionnaire prepared on a Google Form was sent to the persons in charge of each company and their answers were analyzed. Questions were asked about raw materials for packaging materials, display of the recycling symbols on packaging materials, attachment of paper instruction manuals, disposing and recycling of unused artificial joint prosthesis products and old surgical instruments, each company’s efforts to reduce the amount of packaging materials, and the corporate structure and commitments to the SDGs of each company.

Results

Fourteen companies responded to the questionnaires (response rate: 85.7%). Paper, polyethylene, and polyethylene terephthalate were found to be the common packaging materials. The recycle symbol display rates on packaging materials were as low as 14%, except for the plastic containers (43%). Paper instruction manuals enclosed with products are being phased out, and instruction manuals will now be available electronically. Recycling of unused products and their packaging materials at the time of disposal was insufficient. Similarly, recycling of old surgical instruments at disposal was insufficient, as were manufacturers’ efforts to reduce product packaging materials. Only six (43%) and eight companies (57%) were working to reduce the use of paper and plastic packaging materials, respectively. However, some manufacturers have made efforts to design their products to reduce the amount of paper and plastics used as much as possible from the early stage of the product development process. The number of staff in charge of SDGs in Japan is small, whether in Japanese or foreign companies and specialized or integrated enterprises. When the headquarters of foreign companies were included, the number was relatively large. Regarding external consultants and advisors on SDGs, there were none in the country, and only four companies, including those with overseas headquarters, have contracted consultants. Though eight companies disseminated their general sustainability information on their websites, only two companies had web pages describing their current status and future plans for reducing the amount of packaging materials used in prosthetic joint products.

Conclusions

At present, the rate of display of recycling symbols on the packaging materials of joint prosthesis products is low. Although there are differences among manufacturers, the rates of reducing/recycling packaging materials and recycling unused products and old surgical instruments at disposal are generally insufficient. There is little human-resource investment in the SDGs, especially in Japan. Though most companies furnished information on their websites regarding sustainability in general, few companies specifically provided information on the reduction of materials used in product packaging.

## Introduction

Orthopedic implant products, such as artificial joints, fracture fixation devices, and spinal instruments, are essential tools in modern orthopedic surgeries. These implant products are packed in a rigorous fashion to maintain their sterile condition and the quality of the products for a long time. In general, packaging materials include an outer wrapping film, a cardboard box, a plastic container with a seal, a plastic bag for the implant, and additional packing elements to fix or protect the implant. Moreover, more than one plastic containers are used for some artificial joint products.

The field of orthopedics produces 60% more waste on average than any other surgical specialty [1]. Arthroplasty procedures, such as total hip and knee arthroplasties, generate significantly more waste compared to other orthopedic procedures [1-6]. It is not surprising that the more implant products are used in surgery, the higher the amount of packaging material waste. In addition, until recently, it was required by law in many countries that individual products be accompanied by a paper instruction manual. Concerns have been raised about the increase in paper resource waste due to this law. In Japan, the Act on Securing Quality, Efficacy, and Safety of Products Including Pharmaceuticals and Medical Devices mandated orthopedic implant manufacturers to attach paper instruction manuals for individual products, though this law was recently amended [7].

All member countries of the United Nations came together in 2015 and made a historic promise to secure the rights and well-being of everyone on a healthy, thriving planet when they adopted the 2030 Agenda for Sustainable Development and its 17 Sustainable Development Goals (SDGs). Of the 17 goals, goal 12 involves ensuring sustainable consumption and production patterns (responsible consumption and production), which is key to sustaining the livelihoods of current and future generations. The fifth target of goal 12 is as follows: “By 2030, substantially reduce waste generation through prevention, reduction, recycling, and reuse” [8]. The medical community is also becoming increasingly concerned about the impact of medical waste on the global environment, as announced in the 2018 report of the Lancet Countdown [9].

To date, no study has surveyed orthopedic artificial joint manufacturers about recycling and reducing packaging materials of artificial joint products from the viewpoint of SDGs. Moreover, no study has surveyed artificial joint manufacturers on recycling unused products and old surgical instruments. This questionnaire survey aimed to clarify the current status of orthopedic artificial joint manufacturers concerning disposal, recycling, and reduction of packing materials for their products from the viewpoint of SDGs.

## Materials and methods

Sixteen companies that sell orthopedic artificial joint products in Japan were included in the survey: four Japanese and 12 foreign companies. Of the four Japanese companies, one was exclusively engaged in the sale of prostheses, and three were engaged in manufacturing and sales. All 12 foreign companies were engaged in manufacturing and sales.

Using a written request, the survey’s aims were explained to each company’s senior sales personnel. An English translation was sent to headquarters via Japanese personnel for the foreign companies. A Google Form was used to send a questionnaire to the persons in charge of each company and to analyze their answers. Per the Google Form’s question and answer creation method, radio buttons (○) were used to choose one answer from multiple options, and checkboxes (□) were used for choosing multiple answers. Depending on the question, free-text entry was allowed. Although the respondents’ e-mail addresses were collected when the survey was completed, the names of individual responders and their companies were concealed.

**Figure 1:**
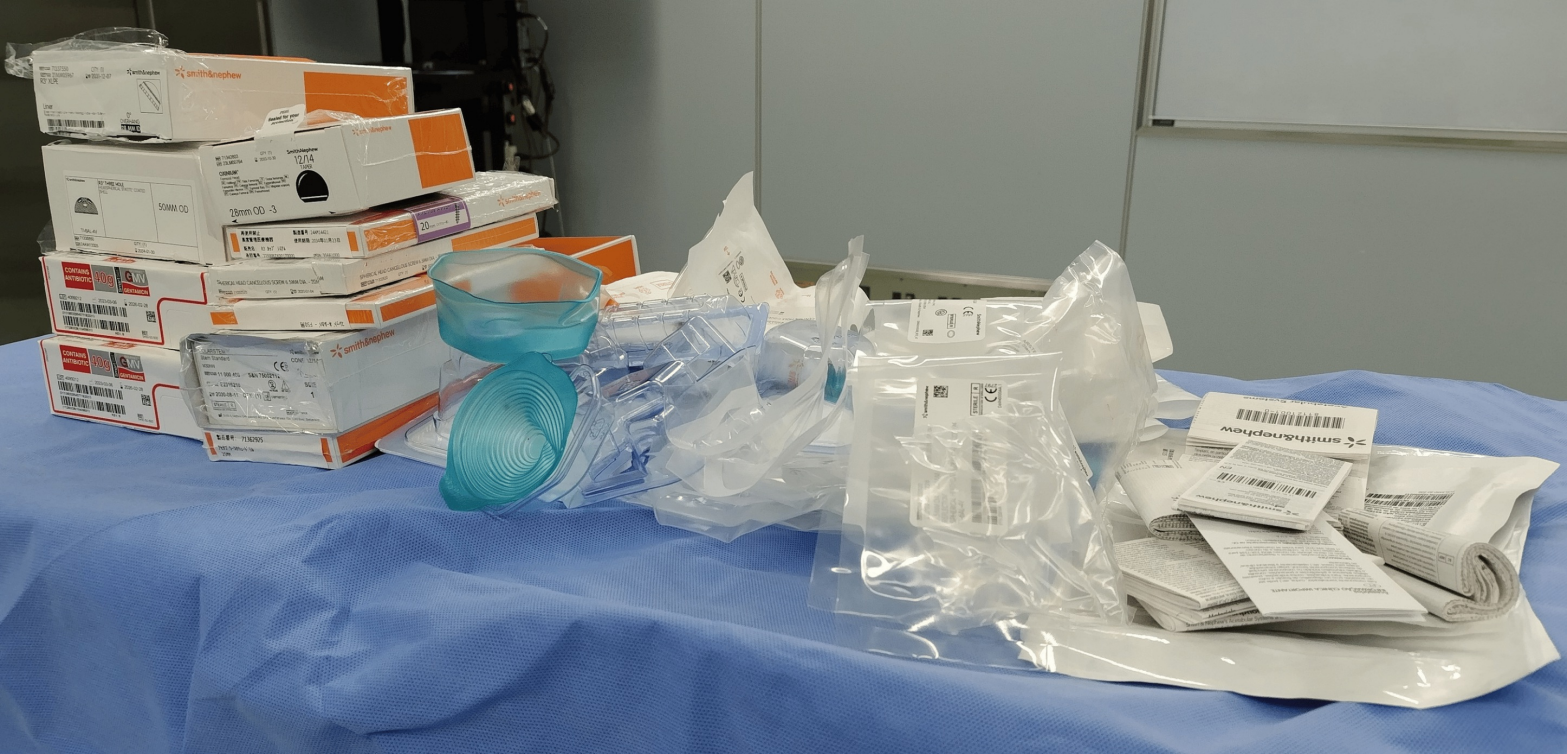
The waste from packaging materials in a single total hip arthroplasty operation From left to right: cardboard boxes with wrapping films, plastic containers with seals, additional packing elements, plastic bags, and paper instruction manuals

**Figure 2:**
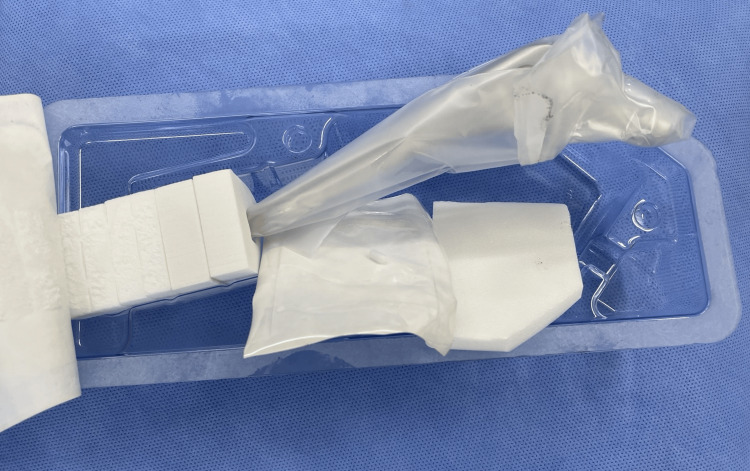
The packaging material of a femoral stem used in total hip arthroplasty The femoral stem is introduced to the surgical field with a plastic container. The femoral stem is contained in a plastic bag. Additional packaging elements are used at the stem end

The survey consisted of six parts. In Part I, respondents were asked about raw materials for packaging materials (Figures 1, 2).

Abbreviations of plastic materials related to this study are listed here, according to the rule of the International Organization for Standardization [10]: polyamide (PA), polyethylene (PE), polyethylene terephthalate (PET), polypropylene (PP), polystyrene (PS), polyurethane (PUR), polyvinyl chloride (PVC), and silicone plastic (SI). Of note, nylon was included in PA, Tyvek was included in PE, and polyethylene terephthalate glycol (PETG) was included in PET in this study.

In Part II, respondents were asked about the display of the recycling symbols on packaging materials. In Part III, respondents were queried about the attachment of paper instruction manuals. In Part IV, respondents were asked about disposing and recycling of unused artificial joint prosthesis products and old surgical instruments. In Part V, respondents were asked about each company’s efforts to reduce the amount of packaging materials. In part VI, respondents were asked about the corporate structure and commitment to the SDGs of each company. In addition, the websites of the companies were visited to investigate their sustainability initiatives. If respondents voluntarily sent a weblink, they were viewed. If not, the author checked whether SDG-related information was available on the company's website.

## Results

Fourteen of 16 companies responded to the questionnaires (response rate: 85.7%). The questions (Q) and the answers provided are presented in detail below.

Part I: Raw materials for packaging materials

Questions 1-6 are presented in Table 1.

**Table 1:** Questions on raw materials for packaging materials ○: Choose one answer from multiple options. □: Choose multiple answers from multiple options PA: polyamide; PE: polyethylene; PET: polyethylene terephthalate; PP: polypropylene; PS: polystyrene; PUR: polyurethane; PVC: polyvinyl chloride; SI: silicone plastic

Questions 1-6										
Q1	Which material do you use for the outer wrapping film to cover the outer box?									
○ PA ○ PE ○ PET ○ PP ○ PS ○ PUR ○ PVC ○ SI										
Q2	Which materials do you use for the plastic containers?									
□ PA □ PE □ PET □ PP □ PS □ PUR □ PVC □ SI										
Q3	Which material do you use most frequently for the plastic containers?									
○ PA ○ PE ○ PET ○ PP ○ PS ○ PUR ○ PVC ○ SI										
Q4	Which materials do you use for the seal?									
□ PE □ PET □ PP □ paper										
Q5	Inside the plastic containers, you may put the implants in plastic bags. Which materials do you use?									
□ PA □ PE □ PET □ PP □ PVC										
Q6	Inside the plastic containers, you may use additional packing elements to protect or secure the implants. Which materials do you use?									
□ PA □ PE □ PET □ PP □ PS □ PUR □ PVC □ SI □ Paper □ Cotton										

**Figure 3:**
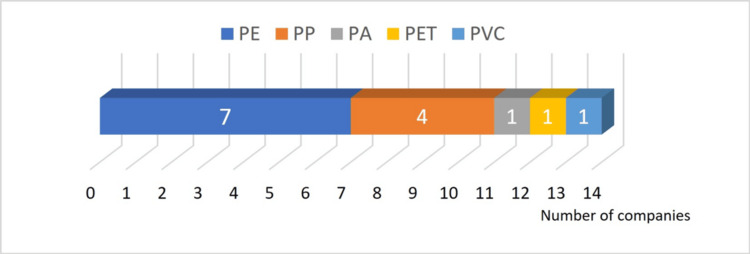
Materials used for the outer wrapping film PA: polyamide; PE: polyethylene; PET: polyethylene terephthalate; PP: polypropylene; PVC: polyvinyl chloride

Answer to Q1: PE was the most used material, while PP was the second most, for the outer wrapping film to cover the outer box (Figure 3).

**Figure 4:**
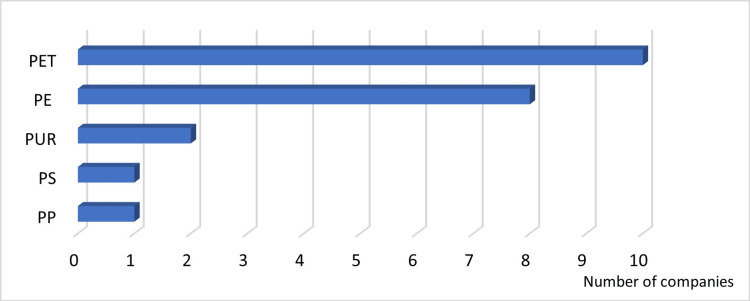
Materials used for plastic containers PE: polyethylene; PET: polyethylene terephthalate; PP: polypropylene; PS: polystyrene; PUR: polyurethane

Answer to Q2: PET and PE were common materials used for plastic containers (Figure 4).

**Figure 5:**
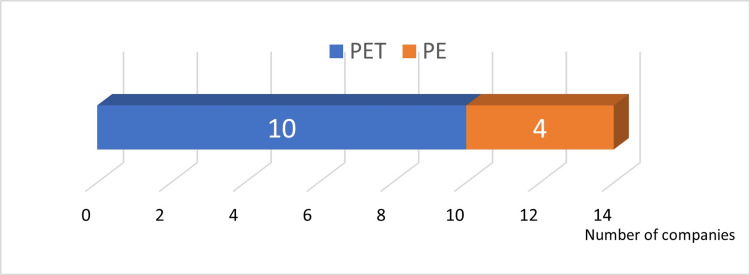
Materials most frequently used for plastic containers PE: polyethylene; PET: polyethylene terephthalate

Answer to Q3: PET was the most frequently used material for plastic containers (Figure 5).

**Figure 6:**
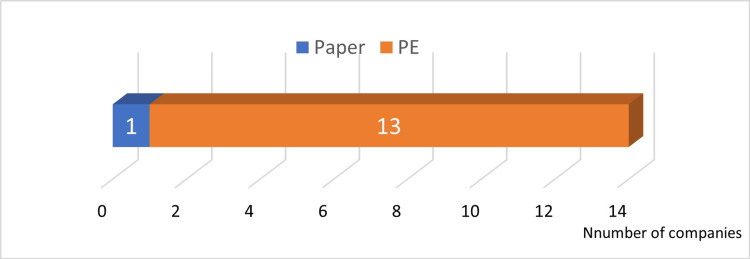
Materials used for the seal PE: polyethylene

Answer to Q4: PE was widely used for the seal (13 companies, 93%; Figure 6).

**Figure 7:**
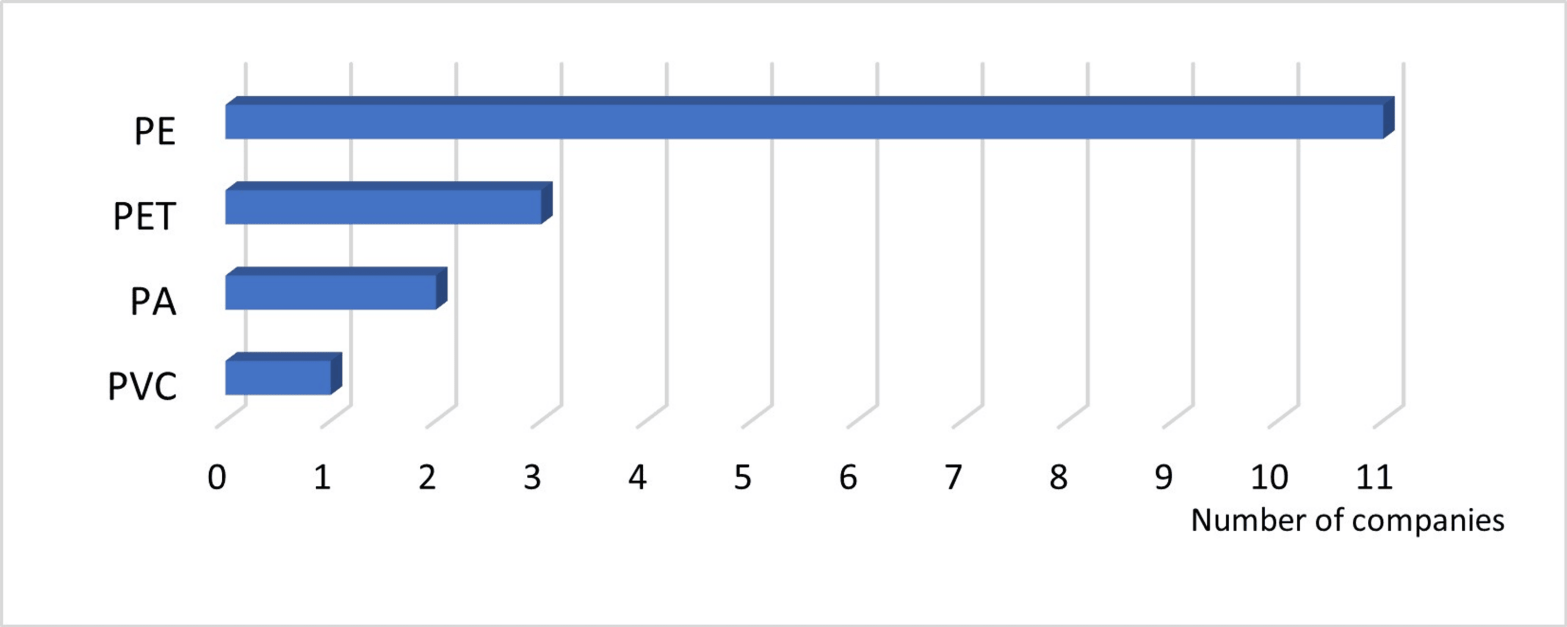
Materials used for plastic bags PA: polyamide; PE: polyethylene; PET: polyethylene terephthalate; PVC: polyvinyl chloride

Answer to Q5: PE was the most frequently used material for the plastic implant bag (Figure 7).

**Figure 8:**
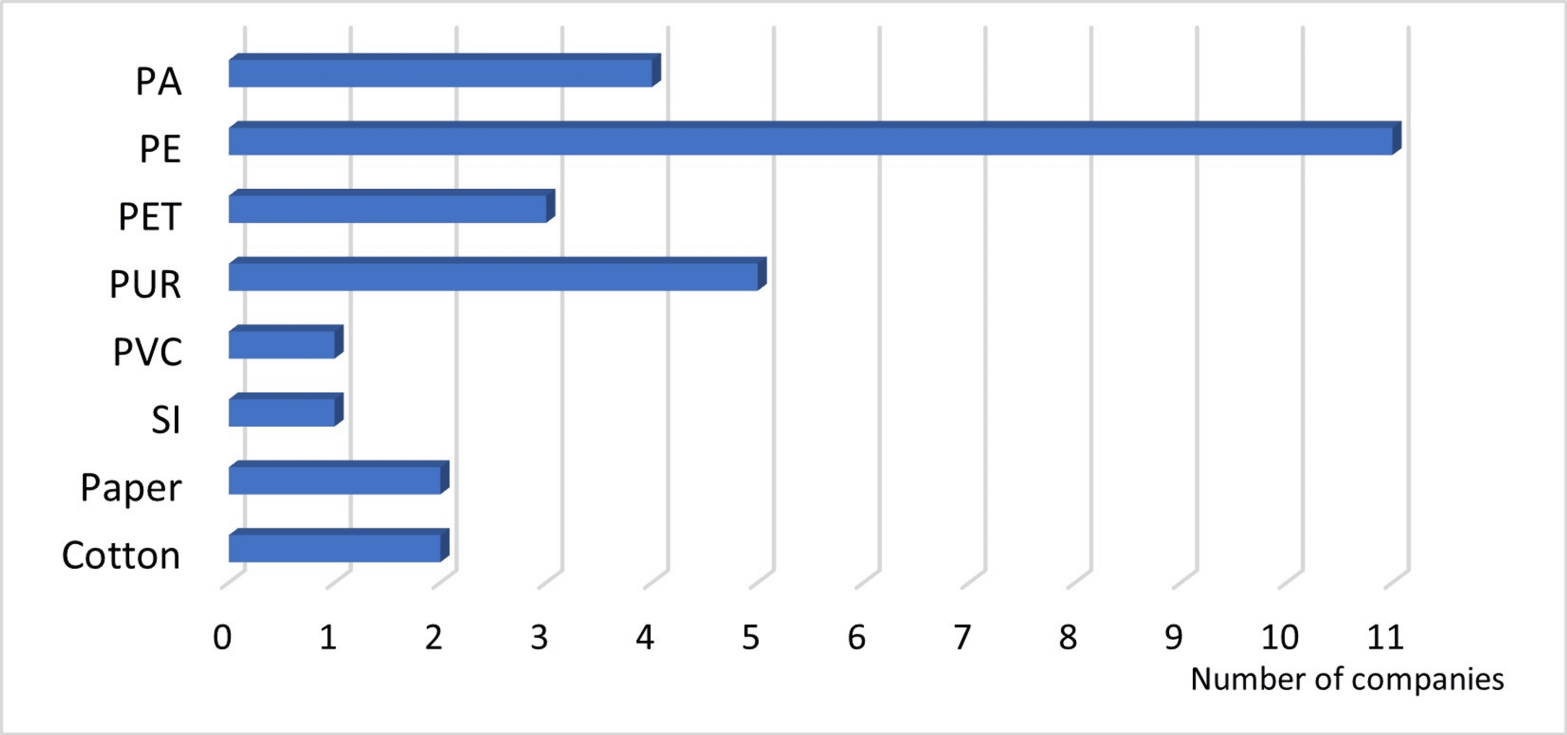
Materials used for additional packing elements PA: polyamide; PE: polyethylene; PET: polyethylene terephthalate; PUR: polyurethane; PVC: polyvinyl chloride; SI: silicone plastic

Answer to Q6: PE was the most commonly used material for the additional packing elements (11 companies); PUR was the second most (five companies), and PA was the third most (four companies) commonly used (Figure 8).

Part II: Display of recycling symbols on packaging materials

Questions 7-16 are presented in Table 2.

**Table 2:** Questions on display of recycling symbols on packaging materials ○: Choose one answer from multiple options

Questions 7-16									
Q7	Do you display a recycling symbol on the outer boxes?								
○ Yes ○ No									
Q8	For those who selected “Yes” in the previous question, how often do you display the recycling symbol on the outer boxes? Please select an applicable answer from the following list								
○ Approximately 1/3 ○ Approximately 1/2 ○ Approximately 2/3 ○ All the outer boxes									
Q9	Do you display a recycling symbol on the plastic containers?								
○ Yes ○ No									
Q10	For those who selected “Yes” in the previous question, how often do you display the recycling symbol on the plastic containers? Please select an applicable answer from the following list.								
○ Approximately 1/3 ○ Approximately 1/2 ○ Approximately 2/3 ○ All the plastic containers									
Q11	Do you display a recycling symbol on the seals?								
○ Yes ○ No									
Q12	For those who selected “Yes” in the previous question, how often do you display the recycling symbol on the seals? Please select an applicable answer from the following list								
○ Approximately 1/3 ○ Approximately 1/2 ○ Approximately 2/3 ○ All the seals									
Q13	Do you display a recycling symbol on the plastic bag?								
○ Yes ○ No									
Q14	For those who selected “Yes” in the previous question, how often do you display the recycling symbol on the plastic bags? Please select an applicable answer from the following list								
○ Approximately 1/3 ○ Approximately 1/2 ○ Approximately 2/3 ○ All the plastic bags									
Q15	Do you display a recycling symbol on the additional packing elements used inside the plastic containers?								
○ Yes ○ No									
Q16	For those who selected “Yes” in the previous question, how often do you display the recycling symbol on the additional packing elements? Please select an applicable answer from the following list								
○ Approximately 1/3 ○ Approximately 1/2 ○ Approximately 2/3 ○ All the additional packing elements									

Answers to Q7 through Q16 are summarized in Table 3. Overall, very few companies displayed the recycling symbol on their packaging materials. Among packaging materials, the recycling symbol was the most frequently displayed on plastic containers (43%).

**Table 3:** Answers to questions 7-16 The number of applicable companies is shown

Type of packaging materials	Recycle symbol displayed		Frequency of display		
	No	Yes	Approx. 1/3	Approx. 2/3	All
Outer box	12	2	1	-	1
Plastic container	8	6	3	2	1
Seal	12	2	-	1	1
Plastic bag	14	0	-	-	-
Additional packing elements	12	2	-	1	1

Part III: Attaching paper instruction manuals

Questions 17 and 18 are presented in Table 4.

**Table 4:** Questions on attachment of paper instruction manuals ○: Choose one answer from multiple options

Questions 17 and 18							
Q17	By law, a paper instruction manual is attached to each product. Are you in favor of abolishing paper instruction manuals and using electronic ones instead?						
○ Yes ○ No							
Q18	Have you already abolished, or are you planning to abolish the paper instruction manuals?						
○ Yes ○ No							

**Figure 9:**
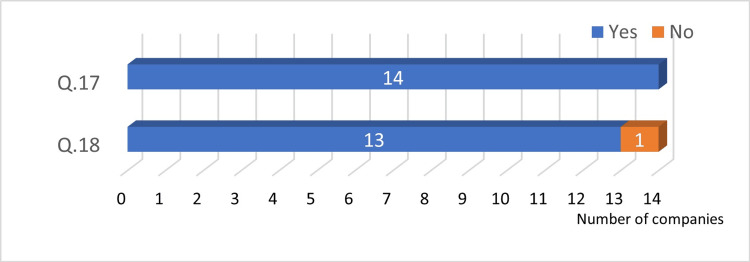
Attaching paper instruction manuals

Answers to Q17 and Q18: All companies are in favor of abolishing paper instruction manuals and using electronic ones instead. At the time of the survey, 11 out of 12 companies had already abolished or started to abolish paper instruction manuals (Figure 9).

Part IV: Disposal and recycling of unused artificial joint prosthesis products and old surgical instruments (excluding single-use items, and including toolboxes)

Questions 19-22 are presented in Table 5.

**Table 5:** Questions on disposal and recycling of unused artificial joint prosthesis products and old surgical instruments ○: Choose one answer from multiple options. □: Choose multiple answers from multiple options

Questions 19-22								
Q19	Do you separate implants and packaging materials according to raw materials (e.g., paper, plastic, metal, and ceramic) at the time of disposal?							
○ Yes ○ No								
Q20	For those who selected “Yes” in the previous question, which materials do you recycle? Please select all applicable answers from the following list							
□ Paper □ Plastics □ Metals □ Ceramics								
Q21	Do you recycle the surgical instruments and toolboxes at the time of disposal?							
○ Yes ○ No								
Q22	For those who selected “Yes” in the previous question, please specify the materials you recycle							

**Figure 10:**
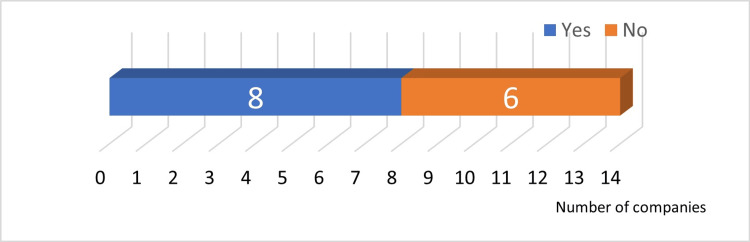
Separation of implants and packaging materials by raw materials

Answer to Q19: Eight companies separated packaging materials and implants by raw material (Figure 10).

**Figure 11:**
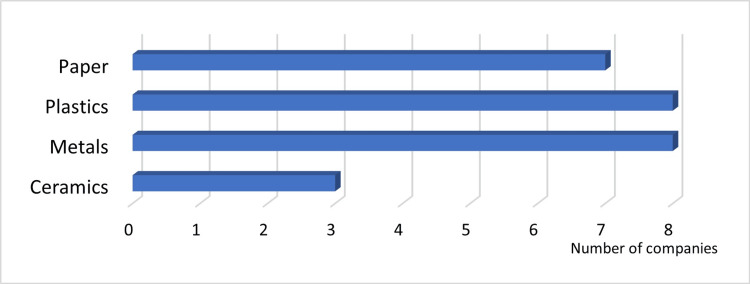
Recycled materials

Answer to Q20: Except for ceramics, recycling rates were high after material separation (Figure 11).

**Figure 12:**
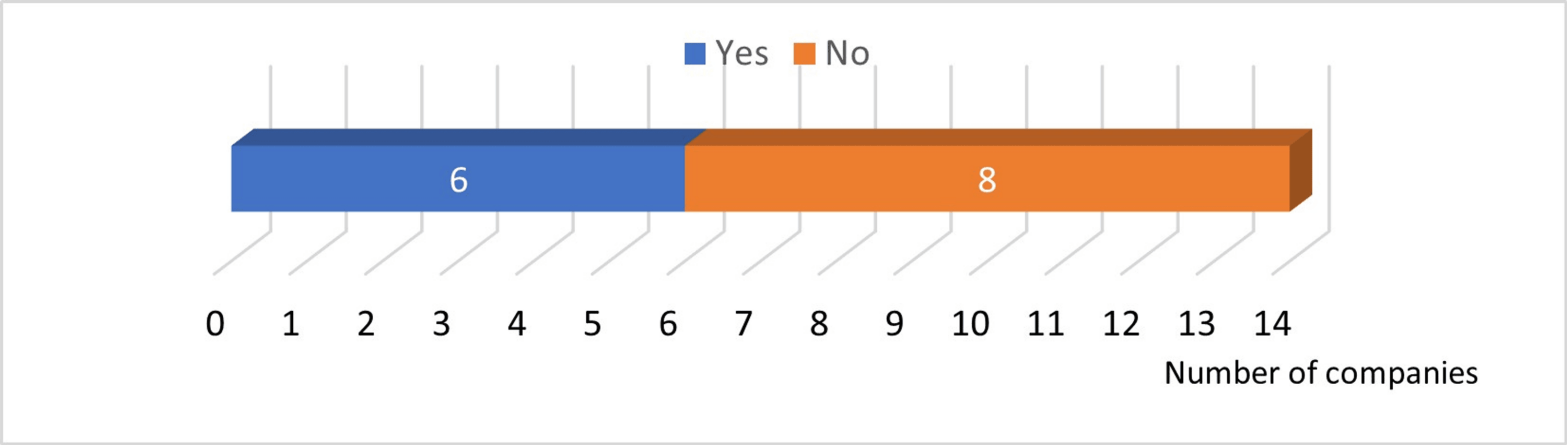
Recycling of old surgical instruments

Answer to Q21: Six companies recycled old surgical instruments and toolboxes (Figure 12).

Answer to Q22: Two companies gave specific answers. One company recycled metals, plastics, rubber, and batteries, and the other recycled stainless-steel alloy, titanium alloy, and aluminum alloy.

Part V: Reduction of implant packaging materials

Questions 23-26 are presented in Table 6.

**Table 6:** Questions on reduction of implant packaging materials ○: Choose one answer from multiple options

Questions 23-26								
Q23	Is your company working to reduce the use of paper packaging materials?							
○ Yes ○ No								
Q24	For those who selected “Yes” in the previous question, please describe your specific reduction methods and plans							
Q25	Is your company working to reduce the use of plastic packaging materials?							
○ Yes ○ No								
Q26	For those who selected “Yes” in the previous question, please describe your specific reduction methods and plans							

**Figure 13:**
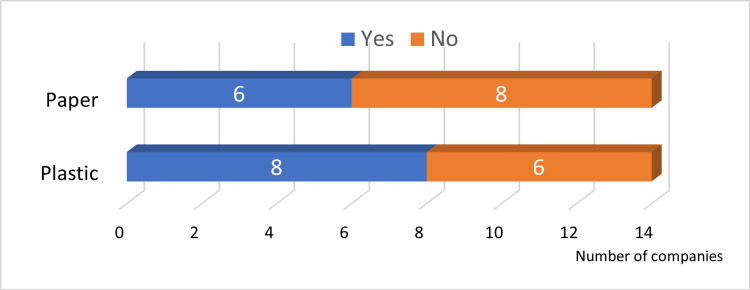
Reduction of implant packaging materials

Answers to Q23 and Q25: Six companies were working to reduce the use of paper, and eight companies were working to reduce plastic packaging materials (Figure 13).

Answer to Q24: Two methods for the reduction of paper packaging materials were presented. One involves reducing paper mass by simplifying and minimizing the package and integrating several products into a common package at an early stage of the product development process. Another is replacing the paper with plastic: replace the cardboard box with recyclable plastic, use Tyvek instead of paper, and use recyclable plastic air cushions instead of cushioning paper.

Answer to Q26: Seven companies presented concrete plans for the reduction of plastic packaging materials. Two companies considered reducing plastic mass at an early stage of the packing specification in parallel to the product development process. Other than that, reduction of the thickness of plastic containers, reduction of the usage of PE, usage of single sterile packing instead of double packing, abolition of wrapping film for cardboard boxes, and replacement of the blister pack with a pouch pack were presented.

Part VI: Corporate structure and commitment to the SDGs of each company

Questions 27-39 are presented in Table 7.

**Table 7:** Questions on corporate structure and commitment to the SDGs of each company ○: Choose one answer from multiple options SDGs: Sustainable Development Goals

Questions 27-39											
Q27	Which of the following corporate structures are applicable to your company?										
	○ A: Japanese company mainly engaged in the manufacture and sale of medical devices										
	○ B: Japanese company that not only engages in the manufacture and sale of medical devices but also expands into other fields										
	○ C: Japanese subsidiary (branch office) of a foreign company mainly engaged in the manufacture and sale of medical devices										
	○ D: Japanese subsidiary (branch office) of a foreign company that not only engages in the manufacture and sale of medical devices but also expands into other fields										
Q28	For those who selected “A” in the previous question, how many staff members are in charge of the SDGs?										
○ 0 persons ○ 1-4 persons ○ 5-9 persons ○ 10 or more persons											
Q29	For those who selected “A” in Q27, do you have any external advisors or consultants regarding the SDGs?										
○ Yes ○ No											
Q30	For those who selected “A” in Q27, do you disseminate information about your SDG initiatives?										
○ Yes ○ No											
Q31	For those who selected “Yes” in the previous question, please write specific details										
Q32	For those who selected “B”, “C”, and “D” in Q27, how many staff members in your medical and sales divisions in Japan are in charge of the SDGs?										
○ 0 persons ○ 1-4 persons ○ 5-9 persons ○ 10 or more persons											
Q33	For those who selected “B”, “C”, and “D” in Q27, how many staff members in your entire company (including the head office in the case of international companies) are in charge of the SDGs?										
○ 0 persons ○ 1-4 persons ○ 5-9 persons ○ 10 or more persons											
Q34	For those who selected “B”, “C”, and “D” in Q27, do you have any external advisors or consultants related to the SDGs in your medical and sales divisions in Japan?										
○ Yes ○ No											
Q35	For those who selected “B”, “C”, and “D” in Q27, does your company as a whole (including the head office in the case of international companies) have any external advisors or consultants on the SDGs?										
○ Yes ○ No											
Q36	For those who selected “B”, “C”, and “D” in Q27, does the medical device manufacturing and sales division in Japan disseminate information on its effort toward the SDGs?										
○ Yes ○ No											
Q37	For those who selected “Yes” in the previous question, please write specific details										
Q38	For those who selected “B”, “C”, and “D” in Q27, does the company as a whole (including the head office in the case of international companies) disseminate information on its SDG initiatives?										
○ Yes ○ No											
Q39	For those who selected “Yes” in the previous question, please write specific details										

**Figure 14:**
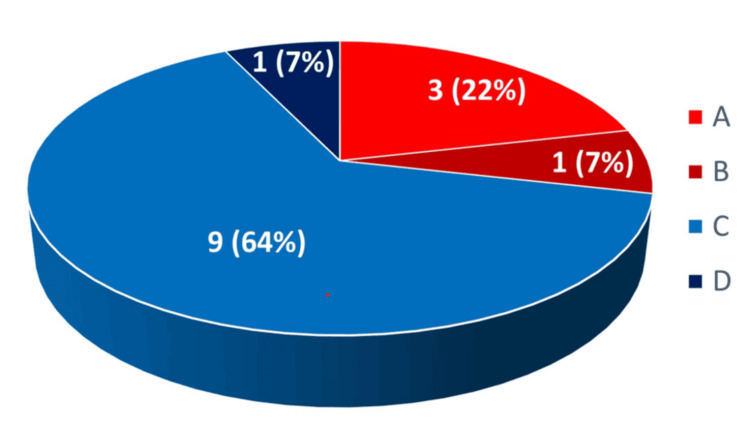
Corporate structure A: Japanese specialized companies (mainly engaged in the manufacture and sale of medical devices). B: Japanese integrated enterprise (not only engages in the manufacture and sale of medical devices but also expands into other fields). C: Foreign specialized companies (mainly engaged in the manufacture and sale of medical devices). D: Foreign integrated enterprise (not only engages in the manufacture and sale of medical devices but also expands into other fields)

The answer to Q27 is shown in Figure 14. There were three Japanese specialized companies (A), one Japanese integrated enterprise (B), nine foreign specialized companies (C), and one foreign integrated enterprise (D).

**Figure 15:**
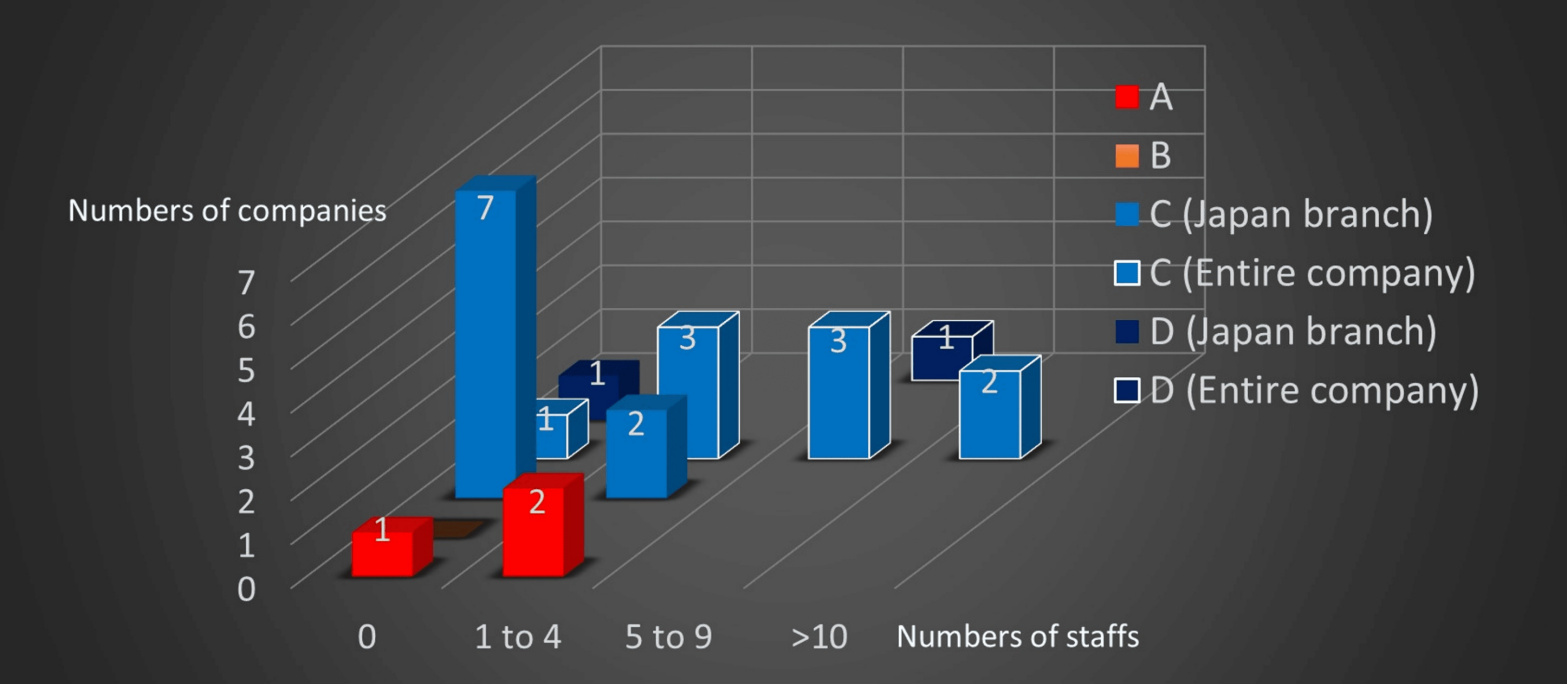
The number of staff in charge of SDGs A: Japanese specialized companies B: Japanese integrated enterprise. C: Foreign specialized companies. D: Foreign integrated enterprise SDGs: Sustainable Development Goals

Answers to Q28, Q32, and Q33 are shown in Figure 15. Of the three Japanese specialized companies (A), one had no designated person while two had one to four persons in charge of SDGs. One Japanese integrated company (B) did not provide an answer to the question. Of the nine foreign specialized companies (C), seven had no one, while two had one to four persons in charge of SDGs in medical and sales divisions in Japan. However, one had no one, three had one to four, three had five to nine, and two had more than 10 persons in charge of SDGs in the entire company. One foreign integrated company (D) had no one in charge of SDGs in the medical and sales divisions in Japan, though it had five to nine persons in the entire company.

Answers to Q29, Q34, and Q35 are shown in Table 8. None of the companies had external advisors or consultants related to SDGs in Japan, regardless of corporate structure. Of the 10 foreign companies, four had external advisors or consultants related to the SDGs in the entire company.

**Table 8:** External advisor or consultant contracts on SDGs The number of applicable companies is shown SDGs: Sustainable Development Goals

	Yes	No	No answer
A: Japanese specialized companies	0	3	-
B: Japanese integrated company	-	-	1
C and D: Foreign companies, Japan branch	0	10	-
C and D: Foreign companies, entire	4	4	2

Answers to Q30, Q31, Q36, Q37, Q38 and Q39 are summarized in Tables 9-10. For Q30, Q36, and Q38, all but one company answered that they disseminate information about their SDG initiatives (Table 9). As for Q31, Q37, and Q39 (specific methods of disseminating information on the SDGs), all responding companies answered that they present the SDG information on their websites. Though two Japanese (A) companies answered that they disseminated information on the SDG initiative on web pages, the general sustainability information was found on the site of only one company. A Japanese (B) company did not answer these questions, though the company disclosed the general sustainability initiatives on web pages (Table 10). Only three of the 10 foreign (C and D) companies answered that their Japanese branch offices disseminate information on the SDGs (Table 9). While eight foreign companies answered that they disseminate information on the SDGs if their overseas headquarters are included (Table 9), four companies disclosed no information on sustainability on their web pages (Table 10) [11-18].

**Table 9:** SDG information dissemination initiatives Figures refer to the number of companies that responded. A: Japanese specialized companies. B: Japanese integrated enterprise. C: Foreign specialized companies. D: Foreign integrated enterprise SDGs: Sustainable Development Goals

	Q30: Japanese company/Q36: foreign company, Japanese branch		Q38: Foreign company as a whole	
	Yes	No	Yes	No
A	2	1	-	-
B	No answer		-	-
C and D	3	7	8	2

**Table 10:** Actual status of sustainability disclosure on each company's website Figures refer to the number of companies that responded. A: Japanese specialized companies. B: Japanese integrated enterprise. C: Foreign specialized companies. D: Foreign integrated enterprise

	Not found	Only general information	Includes information about packaging material management
A	2	1	-
B		1	-
C and D	4	3	3

The remaining six companies disclosed general information on sustainability on their web pages. Additionally, three companies had web pages on packaging materials. One company (C) had a link on its website called “Packaging Environmental Label”, which published details of the raw materials used in all packs of its artificial joint products [19]. Of special note, two companies reported their initiatives in reducing packing materials [20,21]. One company (C) announced that “by 2025, [they would] incorporate at least 30% post-consumer recycled content into all non-sterile packaging materials. By 2025, [they would] incorporate packaging materials from sustainable sources for new packaging parts. [Ensure] recyclability of waste products (e.g. packaging) for the life cycle of the product. Packaging teams have continued to work on incorporating post-consumer recycled content into non-sterile packaging materials and on sourcing more sustainable packaging materials”.

Another company (D) reported their initiatives in reducing the packing materials. They reported their efforts in “minimizing packaging waste through our value chain: Packaging plays a critical role in maintaining the quality and safety of our products throughout our value chain. However, we also evaluated and defined our packaging needs with an intent to minimize packaging volumes while increasing recycled content of packaging and recyclability”. Moreover, in the part on “Product & Packaging Sustainability”, they stated that “We expanded our hospital surgical device recycling program, which was piloted in Germany in 2021, to include eight countries in Europe. The program allows hospitals to recycle specific metal and plastic components from our single-use instruments while digitally capturing and communicating the environmental impact of salvaging materials such as steel, titanium, aluminum, copper, and chrome steel, as well as specific plastics. Using recycled packaging for medical devices, we launched more than 250 product items in post-consumer recycled paperboard material (93%). The new packaging went through extensive testing to confirm that it stands up to our stringent standards for sterility and structural integrity”.

## Discussion

In the present study, we explored practices related to packaging materials used for artificial joint implant products. Paper and plastics are common packaging materials. The usage of paper is mostly limited to the outer box. Though many kinds of plastics are used for packaging materials, PE and PET are the most common. As for plastic containers, PET is the most common, and PE is the second most common. PE was widely used as the seal, plastic bag, and additional packing elements. PUR was sometimes used as additional packing elements.

It was found that not many companies displayed the recycle symbols on the packaging materials. However, one of the “C” companies displayed the recycle symbols on all the packaging material besides the plastic bag. Of the packaging materials, a relatively high proportion of the recycle symbols were seen on the plastic containers (43%, 6 of 14 companies). The display rate of the recycle symbol on the outer box, seal, and additional packaging elements was as low as 14% (2 of 14 companies). No company displayed the recycle symbol on the plastic bag. It might not be easy to display the recycle symbol on the plastic bag and outer wrapping film. To properly segregate all the packaging materials in the operating theater, the recycle symbols with raw material specifications for all the packaging materials should be displayed, e.g., on the outer box in a similar way to food and household goods.

Orthopedic surgery generates more waste than any other type of surgical procedure [1]. Total hip and knee arthroplasty procedures generate the most waste compared to other orthopedic procedures [1-6]. At the same time, arthroplasty surgery produces substantially more recyclable waste per case than the other orthopedic subspecialties [3,4]. Hazardous waste is not recyclable, while non-hazardous waste such as plastics, cardboard, and various wrapping materials is recyclable. However, waste recycling in hospitals has not progressed very far [3], due to several reasons. The classification of waste in hospitals is not always clear, and recyclable waste may be improperly segregated into general or hazardous waste streams [1,2,6,22-24]. According to Prakash et al., only a small percentage of the waste is recycled in hip and knee arthroplasties, which could be improved through increased use of recyclable plastics and clear labeling of items as recyclable by medical suppliers [6]. To properly segregate waste in operating theatres and increase recyclable waste, a clear recycle symbol display on all the packaging materials and education programs for operating room staff in partnership with waste disposal and implant companies are essential [1,3].

In Japan, the Act on Securing Quality, Efficacy, and Safety of Products Including Pharmaceuticals and Medical Devices (Pharmaceuticals and Medical Devices Act) was amended in December 2019. From August 2021 onward, paper instruction manuals that used to be enclosed with products were abolished in principle. Instruction manuals should now be available for browsing electronically [7]. In our study, 13 of 14 companies had already abolished or intended to abolish the paper instruction manuals at the time of the survey.

Recycling unused artificial joint products could be a challenging task for orthopedic implant manufacturers. Eight of 14 companies (57%) separated their unused artificial joint products (i.e., implants and packaging materials) by raw materials at the time of disposal. Eight companies recycled the plastics and metals, seven recycled the paper, and three recycled ceramics. In the past, many artificial joint products were discontinued due to suboptimal clinical results, model changes, and even the dissolution or merger of the companies. Huge numbers of artificial joint products have been distributed worldwide, and unused products may not necessarily be retrieved by their manufacturers. To properly and completely recycle unused products, we recommend three actions. First, a recycling symbol indicating the raw materials of all packaging materials used should be displayed on the outer box of the product. Second, the recycling symbol should be displayed on each packaging material. Third, information on the raw material of the implant itself should be displayed on the outer box.

Recycling old surgical instruments could be another challenging task for orthopedic implant manufacturers. As most surgical instruments are in consumable stores, deteriorated instruments will be discarded. Generally, each joint prosthesis product has dedicated surgical instrument sets for proper insertion of the implant. Hospitals may lease, rent, or purchase surgical instrument sets from manufacturers. Surgical instruments procured on a rental or lease agreement are usually returned to the manufacturer at the end of the contract period. It is unknown whether the eight companies that indicated that they do not recycle surgical instruments collected used surgical instruments from hospitals. Moreover, the dedicated surgical instrument sets become redundant when a joint prosthesis product is discontinued. Thus, it is necessary to collect and recycle old surgical instruments as much as possible. Although six companies (43%) answered that they recycled the surgical instruments at the time of disposal, only two companies specified the materials that they recycled. One company recycled only metals, and another recycled metals, plastics, rubber, and batteries for electric power tools. Implant manufacturers should collect and recycle old surgical instruments as much as possible to reduce waste.

Traditionally, artificial joint products have been somewhat over-packaged due to market demands. For example, double sterile plastic containers have been routinely used for many products. Inside the plastic containers, implant products were fixed with additional packing elements, so that they would not move in the containers, and finally placed in plastic bags. Some manufacturers used plastic protectors for the neck taper of the femoral stem and the prosthetic femoral head. In the present study, manufacturers’ efforts to reduce product packaging materials were found to be insufficient. Only six (43%) and eight companies (57%) were working to reduce the use of paper and plastic packaging materials, respectively.

Fundamentally, the total amount of paper and plastic used for product packaging must be reduced. To achieve this goal, packaging materials should be downsized and simplified. Some manufacturers suggested replacing paper with plastic to reduce the use of paper. Though this method could reduce paper waste, it may increase plastic waste. Because the usage of paper is mostly limited to the outer box, it is necessary to downsize the box and reduce the thickness of the cardboard to the extent that the strength of the box is not compromised. It is more important to reduce the use of plastics. Some manufacturers suggested reducing the thickness of plastic containers, abolishing the outer wrapping film for cardboard boxes, and replacing a blister pack with a pouch pack.

Commendably, some companies have indicated that they are considering a reduction in packaging material from the early stage of the packing specification in parallel with the product development process [12,16,20,21]. Packaging materials released into the surgical field with the implant, such as plastic bags, plastic protectors, and inner plastic containers (in the case of double plastic containers), should be reduced as much as possible because they are treated as hazardous waste. Hazardous waste is not only unrecyclable, but it also causes a variety of problems. Hazardous waste is commonly incinerated at high temperatures, which leads to an increase in greenhouse gases and the generation of toxic gases, resulting in environmental pollution [2,4,22,23]. Furthermore, the disposal costs of high-temperature incineration of hazardous waste are high and place a huge economic burden on hospitals [1,2,4,6,22].

In investigating the SDG initiatives of the artificial joint manufacturing and sales companies, nationality and corporate structure were examined. There were three Japanese and nine foreign companies mainly engaged in the manufacture and sale of medical devices (A and C companies, respectively). In addition, there was one Japanese and one foreign company that not only engaged in the manufacture and sale of medical devices but also was active in other fields (B and D companies, respectively). The former companies are specialized, while the latter are integrated enterprises. It was found that the number of staff in charge of SDGs in Japan, whether Japanese or foreign-owned companies and specialized or integrated enterprises, was small. Nine (one A, seven C, and one D) companies assigned no SDG staff, and four (two A and two C) companies assigned one to four SDG staff in the Japanese office.

In contrast, the number of staff in charge of SDGs, including in the headquarters of foreign companies, was relatively large: two companies had more than 10 persons, four had five to nine persons, three had one to four persons, and one had no one in charge. In addition, unfortunately, the B company did not answer the question. Assuming that many people are employed by these companies, it must be said that there is not much human investment related to the SDGs. Similarly, no company had external advisors or consultants related to the SDGs in Japan, whether Japanese or foreign-owned companies and specialized or integrated enterprises. Even if the headquarters of foreign companies were included, only four (three C and one D) companies had external advisors or consultants related to the SDGs. This may be attributed to the fact that the company size of orthopedic artificial joint manufacturers is not as large as that of other manufacturing industries (e.g., automotive, chemical, and electrical).

The joint prosthesis manufacturers were asked if they were disseminating information on the SDGs in Japan or globally. Though two of the three Japanese (A) companies answered that they disseminated their SDG information, only one company has disseminated their sustainability information on web pages. Eight of 10 companies (C and D) answered that they disseminated their SDG information; however, in reality, only six companies have disseminated their sustainability information on web pages [11-18]. Though four of the six companies had only general sustainability information, two companies had web pages describing their current status and future plans for reducing the amount of packaging materials used in prosthetic joint products. In the future, other companies should also demonstrate their product development stance regarding the reduction of waste generated from joint prosthesis products on web pages.

This study has several limitations. Firstly, not all joint prosthesis manufacturers in the world were surveyed. Secondly, It is unknown whether the respondents correctly identified the plastic materials used in the packaging, and some companies avoided answering certain difficult questions. Third, though we aimed to survey the current status of the orthopedic industry regarding prosthetic product packaging material disposal, recycling, and reduction, we could have posed more appropriate and relevant questions to the manufacturers. Finally, in cases where respondents did not voluntarily send a weblink regarding SDG information dissemination, the authors might have failed to find the appropriate information on the web pages.

## Conclusions

PE and PET are common plastic packaging materials used in artificial joint products. The practice of displaying recycling symbols on the packaging materials of joint prosthesis products was found to be insufficient. Paper instruction manuals that used to be enclosed with products are being phased out. Instruction manuals will now be available for browsing electronically. Although there are differences among manufacturers, there is generally insufficient recycling of implants and packaging materials at the time of disposal of unused products. Similarly, recycling of old surgical instruments at disposal is insufficient. Approximately half of the manufacturers were working to reduce paper and plastic packaging materials. Ideally, product development that minimizes the use of packaging materials should be conducted simultaneously with the development of new joint prosthesis products. There is little human-resource investment in the SDGs, especially in Japan. Though most companies had information on their websites regarding sustainability in general, few companies specifically provided information on the reduction of materials used in product packaging.
